# Fbxw7 haploinsufficiency loses its protection against DNA damage and accelerates MNU-induced gastric carcinogenesis

**DOI:** 10.18632/oncotarget.16800

**Published:** 2017-04-03

**Authors:** Yannan Jiang, Xinming Qi, Xinyu Liu, Jun Zhang, Jun Ji, Zhenggang Zhu, Jin Ren, Yingyan Yu

**Affiliations:** ^1^ Department of Surgery of Shanghai Ruijin Hospital, Shanghai Institute of Digestive Surgery, Shanghai Key Laboratory for Gastric Neoplasms, Shanghai Jiao Tong University School of Medicine, Shanghai, 200025, China; ^2^ Center for Drug Safety Evaluation and Research, Shanghai Institute of Materia Medica, Chinese Academy of Sciences, Shanghai, 201203, China

**Keywords:** Fbxw7, knockout mouse, N-Methyl-N-nitrosourea, gastric cancer, DNA damage

## Abstract

Fbxw7, a subunit of the SCF E3 ubiquitin ligase, recognizes oncoprotein substrates and leads to their proteasomal degradation. Fbxw7 acts as a tumor suppressor via inducing apoptosis and growth arrest in various kinds of tumors. To clarify the initiating role in gastric carcinogenesis as well as the histologic characterization of tumor in Fbxw7 allele haploinsufficient mice, we generated Fbxw7 heterozygous knockout mice (Fbxw7^+/−^) and treated them with chemical carcinogen N-methyl-N-nitrosourea (MNU) at 5–6 weeks of age. We also treated mouse embryo fibroblasts (MEFs) from Fbxw7^+/−^ and Fbxw7^+/+^ mice with MNU and examined cell DNA damage via comet assay. The protein expression of Fbxw7 and its substrate c-Myc from mouse tumors, as well as human tumors sampled from six patients, were detected by Western blot. As results, the tumor incidence was obviously higher in Fbxw7^+/−^ mice (13/20) than in Fbxw7^+/+^ mice (6/20) after 35-week observation. Intestinal metaplasia (*P* = 0.013) and dysplasia (*P* = 0.036) were more severe in Fbxw7^+/−^ mice than in Fbxw7^+/+^ mice. The repair potential of DNA damage was suppressed in MEFs from Fbxw7^+/−^ mice after MNU exposure. Increased c-Myc expression was accompanied by decreased Fbxw7 protein expression in tumor tissues from mouse and patients. In conclusion, Fbxw7 haploinsufficiency increased the risk of gastric carcinogenesis induced by MNU, which is associated with the accumulation of DNA damage as well as c-Myc oncoprotein.

## INTRODUCTION

Gastric cancer is one of the most common cancers worldwide [1]. It is thought to result from a combined attack by environmental factors and genetic alterations [2]. Activation of oncogenes and silence of tumor suppressors play important roles in gastric carcinogenesis. Fbxw7, a member of the F-box family which is a component of the SKIP-CUL1-F-box protein (SCF) E3 ubiquitin ligase complex, mediates the recognition and binding of its substrate proteins, leading to their K48 ubiquitination and proteasomal degradation [3]. The substrates of Fbxw7 include c-Myc, CyclinE, c-Jun, mTOR, KLF5, Notch1 and Notch4 [4, 5]. Most of them have been shown as oncogenes in multiple types of human cancers. Recent studies revealed that Fbxw7 regulates apoptosis, growth arrest, and epithelial-to-mesenchymal transition in gastric cancer [6]. A reduced expression of Fbxw7 is detected in gastric tumors compared to the paired non-neoplastic specimens and is associated with a highly invasive phenotype in gastric cancer cell lines [7, 8]. In general, Fbxw7 displays a mutation frequency about 6% in multiple human cancers, including gastric cancer [9–11]. Genetic inactivation of Fbxw7 causes chromosomal instability characterized by aneuploidy and micronucleus formation [12] and is associated with the increased rate of radiation-induced tumors [13]. FBXW7 promotes ubiquitination of XRCC4 via lysine 63 linkage to facilitate nonhomologous end-joining, rather than proceeds to XRCC4 degradation [14], while these two pathways of ubiquitination (via K48 and K63 linkage) contribute to the tumor suppressor role of FBXW7 together.

Haploinsufficiency refers to a phenotype in which a diploid cell lost one allele of a gene, and the remaining copy of the gene cannot provide sufficient gene function to maintain the cell phenotype. Haploinsufficiency means a situation for tumor suppression in which functional loss of one allele results in a selective acceleration for tumor growth [15]. Loss of function of tumor suppressors Pten, Dicer1, and Cdkn1b was associated with haploinsufficiency in animal experiments [16–19]. One copy deletion of Fbxw7 gene with a frequency of 45.5% was observed in gastric tumors [8]. Both Tetzlaff, *et al*. and Tsunematsu, *et al*. found that homozygous loss of Fbxw7 led to embryonic death in mice due to the abnormality of heart and vessels, while heterozygous mutation appeared normal and did not develop spontaneous tumors up to 12 months [20, 21]. Mao, *et al*. crossed Fbxw7 heterozygous knockout mice with p53 knockout mice and found that loss of one allele of Fbxw7 increased the lymphoma formation in p53 knockout mice [13]. Fbxw7^+/−^ heterozygous mutation also increased tumors in APC^min/+^ mice and decreased the survival rate [22]. Thus, Fbxw7 haploinsufficiency is related to the increased tumor incidence in animal experiments.

N-methyl-N-nitrosourea (MNU), a chemical carcinogen, has been widely used in gastric carcinogenesis study on mouse models [23, 24]. In our study, we treated Fbxw7 systematically knockout mice with MNU, and investigated the impact of Fbxw7 haploinsufficiency on gastric carcinogenesis. We also analyzed the possible attacking mechanisms of Fbxw7 dysfunction on murine somatic cells.

## RESULTS

### Construction of Fbxw7 knockout mice

**Figure 1:**
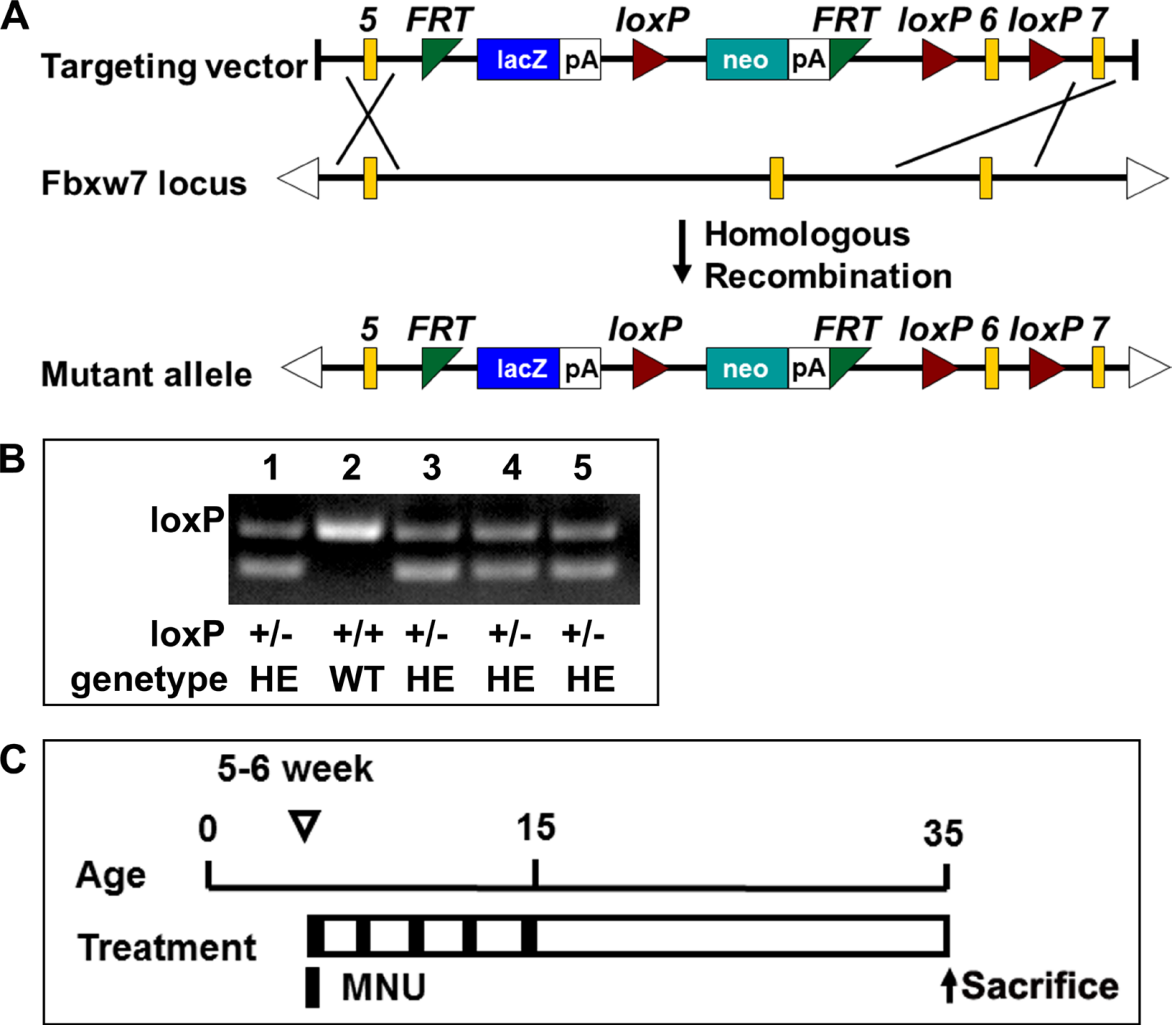
Fbxw7 knockout mice construction and treatment with MNU (**A**) Schematic diagram of the creating Fbxw7 heterozygous knockout mice. (**B**) Genotyping using mouse tail DNA at the age of 2 weeks. PCR screening for the mutant allele revealed a band of 232bp for the wild type allele and 359bp for the mutant allele. (**C)** Experimental design of MNU treatment. The 5–6 weeks old mice were given MNU 240 mg/L in drinking water, every other week for a total exposure of 5 weeks.

The Fbxw7^βgeo^ ES cell (a flexible gene-trap knockout-first, lacZ tagged insertion Fbxw7 allele) was obtained from EUCOMM for generating Fbxw7^βgeo^ mice. These ES clones had passed all rigorous quality control tests of the EUCOMM. Due to homologous recombination, the wild-type allele was replaced by Fbxw7^βgeo^ allele whose gene function would be ablated by a polyadenylation (polyA) signal-mediated transcriptional stop at the end of the lacZ expression marker gene. Fbxw7^βgeo/+^ mice maintained on C57BL/6 genetic background (Figure 1A). Genotyping analysis of the newborn offsprings indicated that Fbxw7^βgeo/+^ and Fbxw7^+/+^ mice were alive, while Fbxw7^βgeo/βgeo^ mice died in embryonic stage, which is consistent with previous reports [20, 21]. The product of PCR with two bands at 232bp and 359bp represents the heterozygous genotype, and product with one band at 359bp represents the wild-type one (Figure 1B). Fbxw7^βgeo/+^ were systemically heterozygous knockout mice (Fbxw7^+/−^). Fbxw7^+/+^ were wild-type mice. 40 mice were used in gastric carcinogenesis study (20 Fbxw7^+/−^ mice and 20 Fbxw7^+/+^ mice, Figure 1C).

### Increased gastric cancer susceptibility of Fbxw7^+/−^ mice under MNU treatment

**Figure 2:**
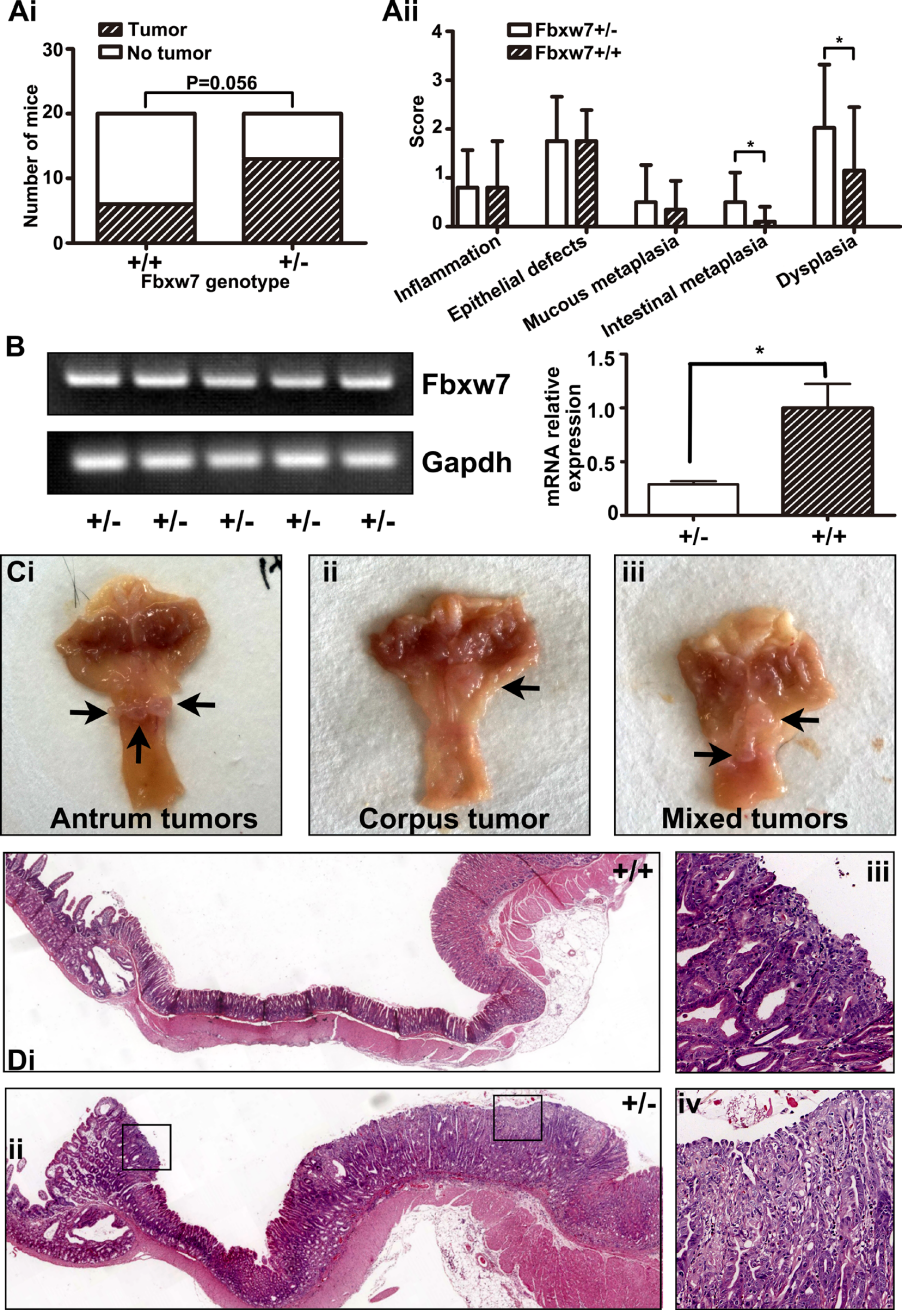
Induction of gastric tumor development in the antrum in Fbxw7^+/−^ mice (**A**) Incidence (Ai) and pathological score (Aii) of gastric tumor formation in Fbxw7^+/−^ and Fbxw7^+/+^ mice at the age of 35 weeks with MNU-based treatment (*n* = 20 each genotype). (**B**) mRNA expression of Fbxw7 from tumors of Fbxw7^+/−^ mice (*n* = 5 each genotype, left figure is from the semi-quantitative examination, and right figure is from the real-time RT-PCR assay). (**C**) Gross morphology of visible tumors (black arrows) in the gastric antrum (Ci), corpus (Cii), or both (“mixed”, Ciii) in Fbxw7^+/−^ mice enlarged. (**D**) Representative photographs of macroscopic views of the entire gastric mucosa in Fbxw7^+/+^ (Di) and Fbxw7^+/−^ (Dii) mice and two microscopic view of an gastric tumor in the Fbxw7^+/−^ mouse (Diii, Div). The 2-tailed *X*^2^ test was used to determine the significance of the tumor incidence. The non-parametric test was used to determine the significance of the difference between pathological scores of each group. “*” refers to statistically significant (*P* < 0.05).

The mice of experimental group (Fbxw7^+/−^) and control group (Fbxw7^+/+^) were treated with MNU in drinking water for 5 weeks, and sacrificed at 35 weeks. No death or weakness was observed. We found no abnormality of mice in chest and abdominal cavity autopsy. Then we cut the stomach along the greater curvature and recorded the tumors. The incidence of gastric tumors in Fbxw7^+/−^ mice was much higher than in wild-type mice (13/20 vs 6/20, *P* = 0.056, Figure 2A). Slides of stomach tissues were stained by hematoxylin-eosin reagent and scored for pathology on a scale of 0 to 4, as previously described [25]. The standard for pathology score was listed on Table 1. The results showed more severe intestinal metaplasia and dysplasia in Fbxw7^+/−^ mice than in wild-type mice (*P* = 0.013 and *P* = 0.036, separately, Figure 2Aii). We tested the mRNA expression of Fbxw7 in tumors. All of the tumors from Fbxw7^+/−^ mice expressed Fbxw7, though the expression levels were lower than controls (*P* = 0.0135) (Figure 2B). The results suggested that the lower expression of Fbxw7 and the higher incidence of gastric cancer were attributed to the Fbxw7 haploinsufficiency. We noticed that most gastric tumors were located in the antrum (antrum vs corpus equals to 16/19 vs 2/19, remaining 1/19 was mixed location, Figure 2C). All of the gastric tumors induced by MNU were intestinal-type carcinoma.

**Table 1:** Murine gastric histopathology scoring paradigm

Parameters	Score				
	0	1	2	3	4
Inflammation	no inflammatory cells	inflammatory cells infiltration of the submucosa with or without infiltration at the very base of the mucosa	inflammatory cells infiltration of the submucosa and the bottom half of the mucosa	inflammatory cells infiltration of the submucosa and greater than 50% of the mucosa	transmural infiltration of inflammatory cells
Epithelial defects	no visible parietal cell and chief cell loss	25% parietal cell loss and 50% chief cell loss	50% parietal cell loss and greater than 75% chief cell loss	75% parietal cell loss and 100% chief cell loss	greater than 75% parietal cell loss and no chief cells
Mucous metaplasia	no visible mucus metaplasia	small foci were present	up to one-third of the parietal cells was affected	two thirds of the parietal cells were affected	greater than two thirds of the parietal cells were affected
Intestinal metaplasia	no visible mucus metaplasia	small foci were present	up to one-third of the corpus was affected	two thirds of the corpus were affected	greater than two thirds of the corpus were affected
Dysplasia	no visible dysplasia	appearing aberrant crypt foci including distortion of normal columnar orientation, increased diameter, asymmetrical cell piling, and back-to-back forms	there is glandular infolding, branching, and more advanced cellular atypia such as increased nuclear-cytoplasmic ratio	cellular distortion with haphazard arrangements, the lesion developed to carcinoma *in situ*	highly dysplastic glands invade into the submucosa or beyond, such as deeper layers, vessels and lymphatics

### Decreased apoptosis of gastric mucosa in Fbxw7^+/−^ mice

**Figure 3:**
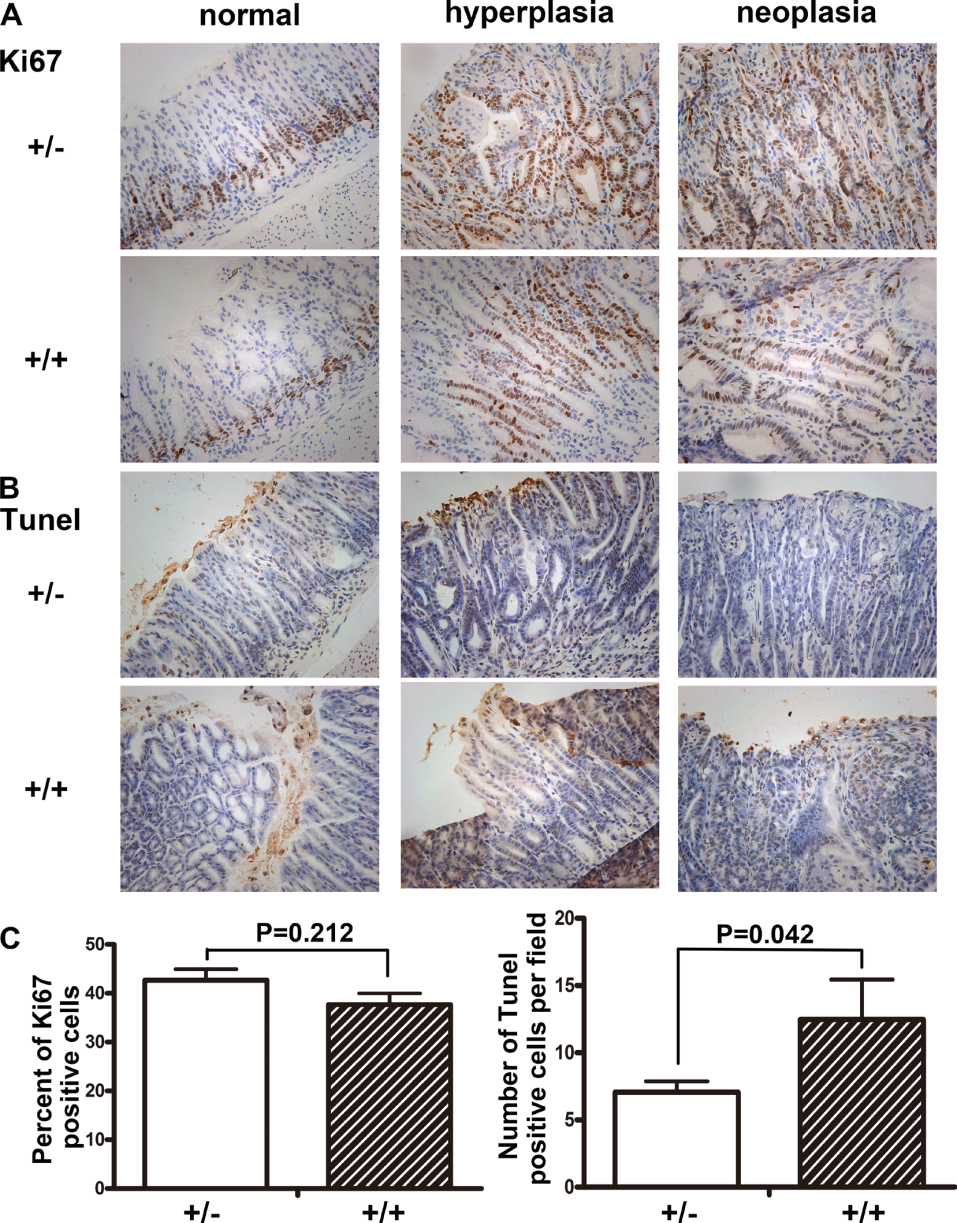
Immunohistochemistry staining of Ki67 and TUNEL in antrum Sections of antral tissues were prepared from Fbxw7^+/−^ and Fbxw7^+/+^ mice. From left to right were: normal, hyperplastic and neoplastic tissues. Cell proliferation was determined by Ki67 staining. Cell apoptosis was determined by TUNEL. The *X*^2^ or *T* test was used to determine the significance of Ki67 and TUNEL studies.

We compared the apoptosis of gastric mucosa by TUNEL assays, and found the apoptotic bodies per field in Fbxw7^+/−^ mice were much less than in Fbxw7^+/+^ mice (*P* = 0.042). Apoptotic bodies of wild-type mice could be found in the mucosal glands as well as in the exfoliated epithelial cell masses (Figure 3B, 3C). The growth activity of gastric mucosa was assayed by Ki67 labeling. There was no significant difference in percent of Ki67 positive cells between Fbxw7^+/−^ mice and controls. Ki67 positive cells were mainly observed in the bottom of crypts of non-cancerous regions, while increased Ki67 positive cells were observed in dysplastic or cancerous regions (Figure 3A, 3C).

### Delayed DNA damage repair in MEFs from Fbxw7^+/−^ mice

**Figure 4:**
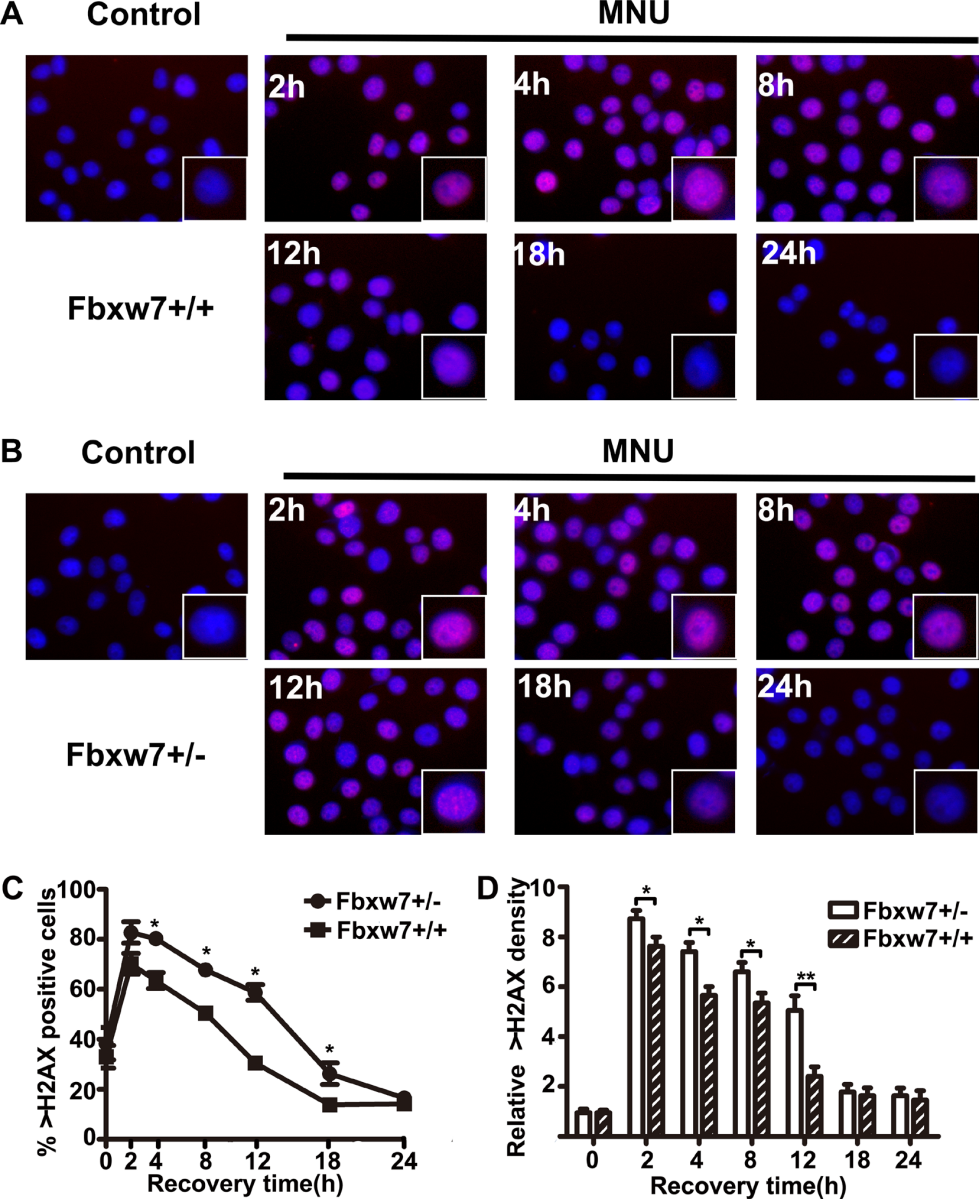
Bimodal pattern of H2AX phosphorylation after treatment with MNU (**A**, **B**) pH2AX foci in MEFs from Fbxw7^+/+^ (A) and Fbxw7^+/−^ (B) after MNU treatment. The cells were collected and fixed at the indicated time points after treatment. (**C**, **D**) The dynamics of pH2AX foci formation after treatment as shown in (A) and (B). Data are presented as mean ± SD, from three independent experiments. The *X*^2^ or *T* test was used to determine the significance of pH2AX positive incidence and intensity. “*” refers to statistically significant (*P* < 0.05).

**Figure 5:**
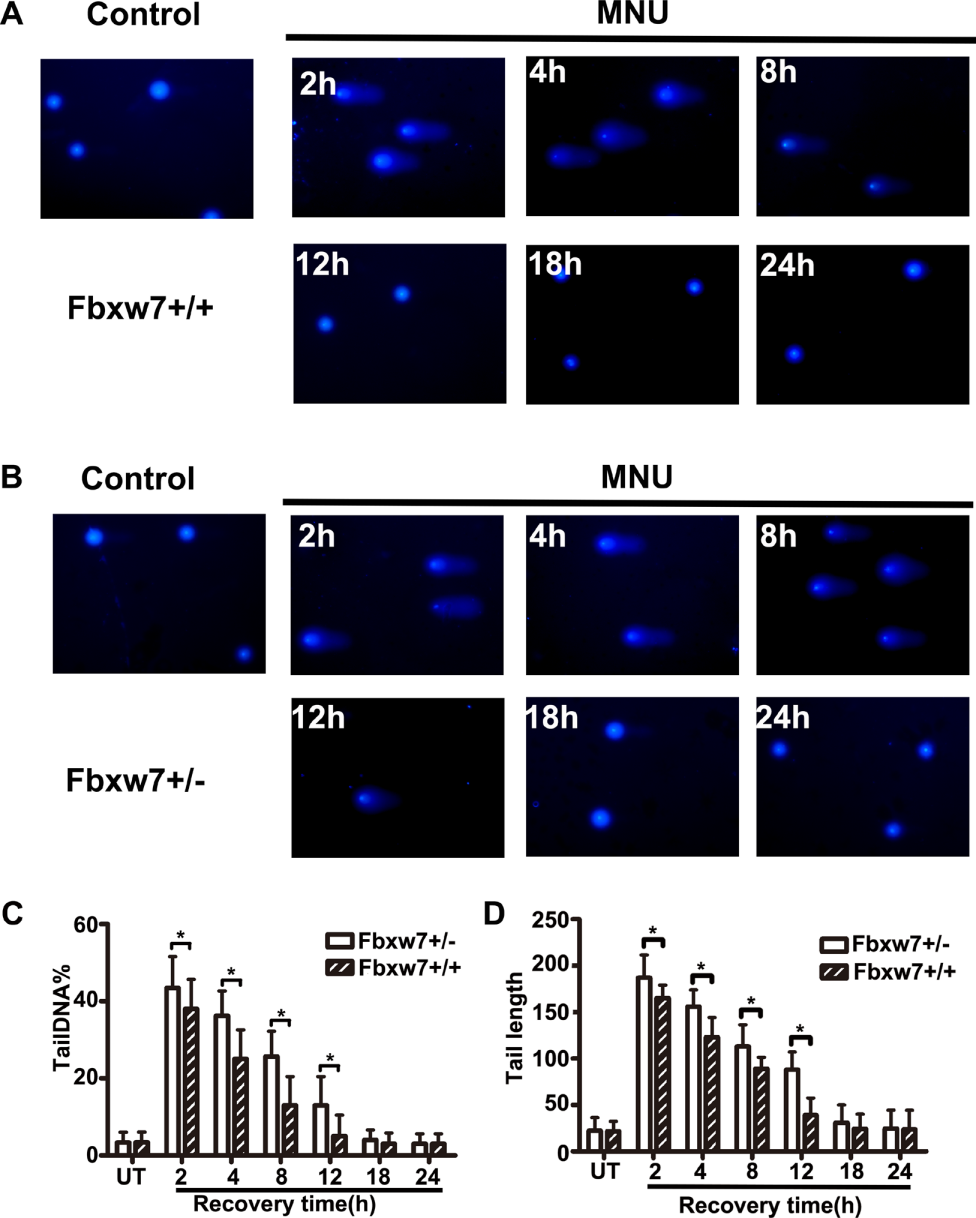
Detection of DNA damage by comet assay (**A**, **B**) The comet images of MEFs from Fbxw7^+/+^ (A) and Fbxw7^+/−^ (B) at given time after MNU treatment. (**C**, **D**) The repair kinetics of DNA damage, which were expressed as the comets tailDNA% (C) and tail length (D). The data are the means and standard deviation from three experiments, and more than 50 comets were analyzed in each experiment. The *T* test was used to determine the significance of comet assay. “*” refers to statistically significant (*P* < 0.05).

**Figure 6:**
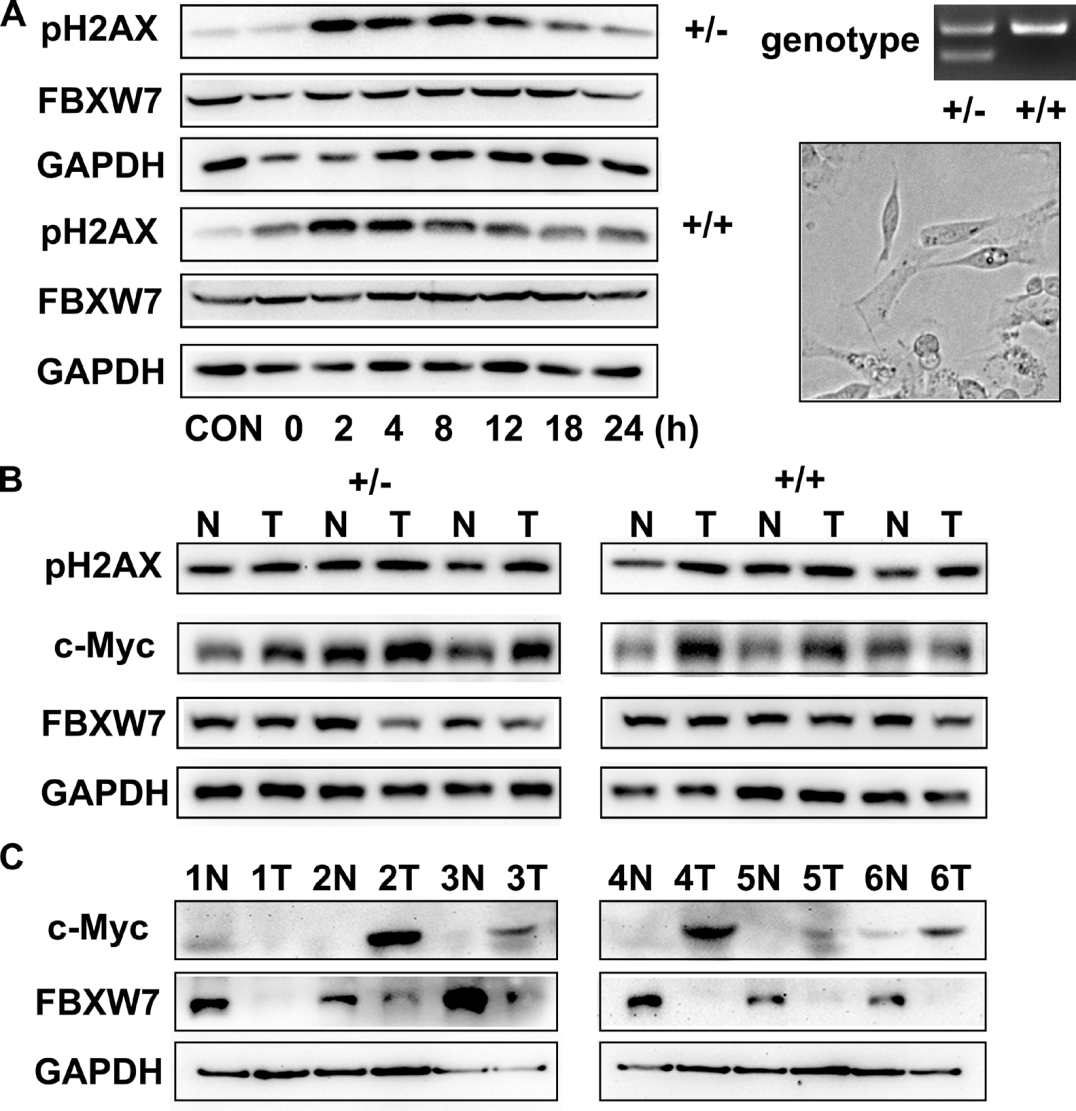
Loss of Fbxw7 expression and overexpression of c-Myc in gastric cancer (**A**) Western blot for pH2AX expression at indicated time points after MNU treatment (left). Genotyping and microscopic view of Fbxw7^+/+^ and Fbxw7^+/−^ MEFs (right). (**B**, **C**) Western blot analyses were performed using total protein lysates extracted from antral mucosa from mice (B) and from patients (C).

To investigate the repair ability of DNA damage after MNU exposure, we assayed pH2AX expression on MEFs from Fbxw7^+/−^ and Fbxw7^+/+^ mice by immunofluorescence. The percent of pH2AX positive cells and relative density of fluorescence reached a peak at 2h post MNU exposure and then decreased gradually in both genotypes. However, the percent of pH2AX positive cells (*P <* 0.05 at 4 h, 8 h, 12 h and 18 h) and relative density (*P <* 0.05 at 4 h, 8 h and 12 h) of fluorescence declined more slowly in Fbxw7^+/−^ MEFs than in wild-type MEFs (Figure 4). We also conducted the comet assay to examine the DNA damage repair in these two MEFs. The results revealed that DNA strand breaks were repaired at 12 h in wild-type MEFs, while the repair was not complete until 18 h in Fbxw7^+/−^ MEFs. Both tailDNA% and tail length were significantly different at 2 h to 12 h (*P <* 0.05) (Figure 5). Western blotting of pH2AX showed similar trends (Figure 6A). The microscopic view and results of genotyping examination of MEFs were presented in Figure 6A.

### Upregulated c-MYC expression in gastric tumors

Since c-MYC protein is degraded via FBXW7-mediated ubiquitin pathway, we examined the protein expression of FBXW7 and c-MYC in gastric tumors and paired normal tissues from six patients (Figure 6C) and mouse models (Figure 6B). We found that the protein expression pattern of FBXW7 and c-MYC were reversely correlated. The decreased FBXW7 expression was accompanied by increased c-MYC expression in cancerous tissues, compared to corresponding normal tissues.

## DISCUSSION

Fbxw7 gene alteration has been found in a variety of tumors, such as lymphoma, glioma, colorectal cancer and gastric cancer [12, 13, 26, 27]. Recently, several genomic sequencing on gastric cancer revealed that the mutant frequency of Fbxw7 gene was about 6–9%, which further suggested the possible role of Fbxw7 gene on gastric carcinogenesis [9, 10, 28–30]. In order to clarify the function of Fbxw7 gene, we constructed Fbxw7 knockout mice. According to literatures, homozygous loss of Fbxw7 is an embryonic dead event, while heterozygous mice show normal phenotype without spontaneous tumors. But the accelerated tumorigenesis had been noticed in lymphoma and colon cancer in Fbxw7 heterozygous mice combined with loss of other tumor suppressors [13, 22]. We were interested in whether haploid loss of Fbxw7 would increase the gastric carcinogenesis. So we treated the Fbxw7 heterozygous knockout mice with chemical carcinogen MNU. By our research, Fbxw7 heterozygotes had higher susceptibilities to gastric cancer after MNU exposure, compared to wild-type mice. Fbxw7 expression was reduced in Fbxw7^+/−^ mice than in controls, but none of tumors from Fbxw7^+/−^ mice showed lost Fbxw7 expression, which meant all mice maintained Fbxw7 expression of the retained one allele. Our results were consistent with previous reports on other types of cancer [13, 31, 32]. The results support that Fbxw7 haploinsufficiency increases the gastric cancer risk. Then we used Ki67 staining and TUNEL assay to examine the proliferation and apoptosis in tumors from Fbxw7^+/−^ mice. Our results showed that cell apoptosis was significantly decreased in tumors from Fbxw7^+/−^ mice, consistent with previous reports that Fbxw7 overexpression induced apoptosis [6].

**Figure 7:**
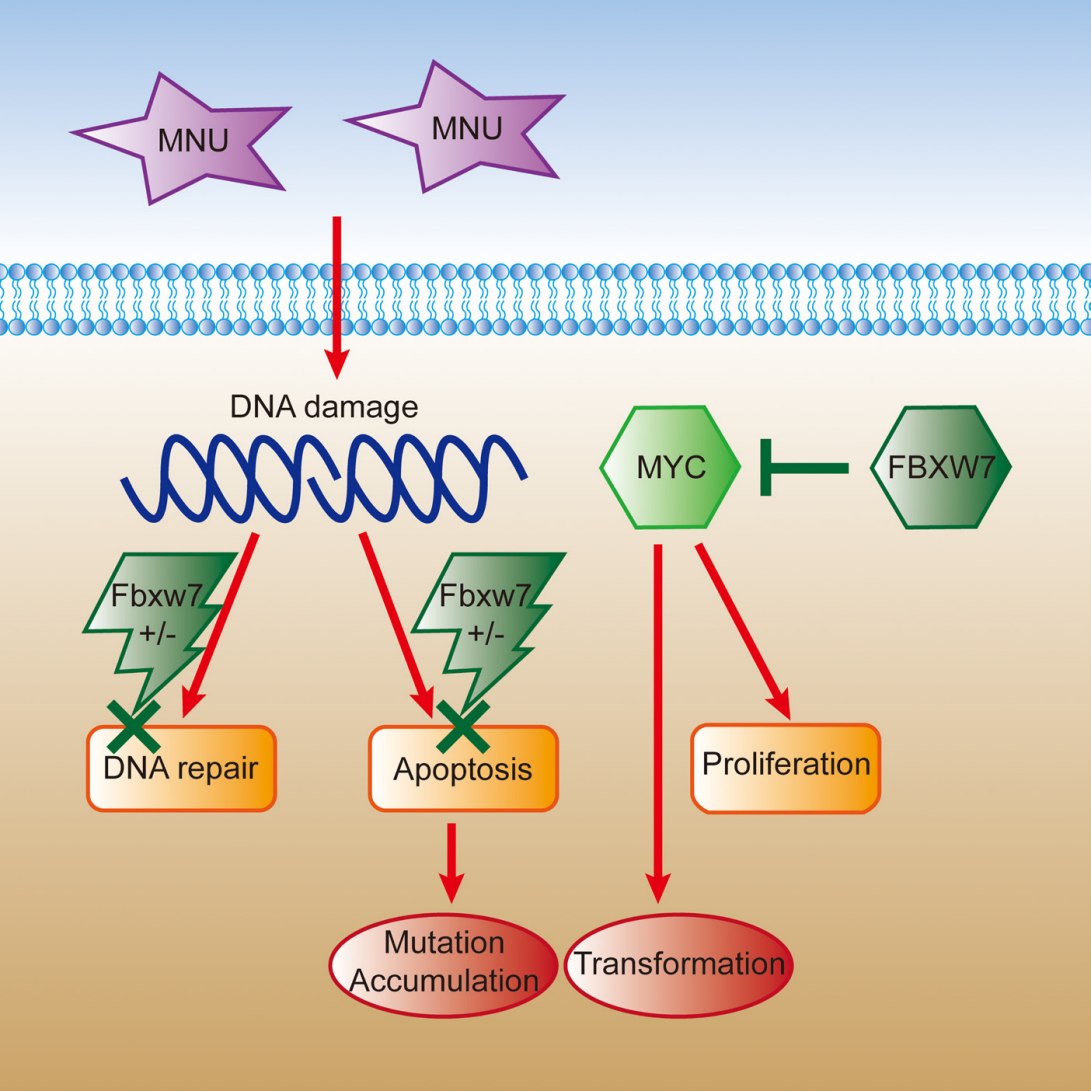
The schematic figure of somatic cells transformation induced by MNU in Fbxw7 haploinsufficiency condition Cell DNA is damaged under the MNU exposure. Due to the haploinsufficiency of Fbxw7, it is difficult to repair mutated DNA as well as proceed apoptosis. Meanwhile, insufficient FBXW7 level results in elevated downstream c-MYC expression and promoted cell transformation and proliferation.

To understand the molecular mechanisms of Fbxw7 haploinsufficiency on gastric carcinogenesis, we examined the expression of oncoprotein c-Myc, which had been noticed as a substrate of FBXW7 protein [7, 8, 33]. We compared the protein expression levels of Fbxw7 and c-Myc and confirmed the reverse expressing relation of these two proteins by Western blot. The protein level of Fbxw7 expression was lower in Fbxw7^+/−^ mice, compared with controls, while c-Myc showed the opposite change, especially in tumors. Fbxw7 haploinsufficiency resulted in decreased Fbxw7 expression and increased c-Myc expression. Considering the role of c-Myc in promoting cell transformation, this may be one of the reasons why Fbxw7^+/−^mice developed more gastric tumors than controls (Figure 7).

Fbxw7 has been reported to be related with DNA damage repair and play its role via K63 linkage, which is completely different from ubiquitin protease degradation system. In order to clarify the role of Fbxw7 haploinsufficiency in gastric carcinogenesis, we used MEFs from Fbxw7^+/−^ and Fbxw7^+/+^ mice to examine the reaction of these two cells to MNU treatment. With the development of genetically engineered mice, MEFs from specific gene mutated mice (deletion of one allele or both) have been cultured and used to study the impacts of the heterozygous or null mutated gene. Mao, *et al*. subjected MEFs from p53^+/−^, p53^−/−^ and wild-type mice to γ-radiation and examined whether activation of p53 by radiation might have an effect on the expression of Fbxw7. They found the increase of Fbxw7 mRNA was dependent on p53 activation [13]. Dai, *et al*. used MEFs from BubR1^+/−^ mice to determine the expression of BubR1 and its activation after microtubule disruption, and found that ablation of one BubR1 allele reduced the expression and activation of the other allele [34]. All above-mentioned studies demonstrate that MEFs originated from gene knockout mice lacking one or both alleles at DNA levels have unique advantages in *in-vitro* research, especially in heterozygous mutation study. So we analyzed the effect of Fbxw7 haploinsufficiency on cultured MEFs from Fbxw7^+/−^ and Fbxw7^+/+^ mice after MNU exposure.

MNU is one of the alkylating agents and widely used in gastric carcinogenesis study [35]. MNU directly alkylates nitrogen and oxygen atoms of DNA bases, resulting in single nucleotide mutations and DNA strand breaks [36]. Regarding to DNA damage, H2AX will be recruited and phosphorylated at DNA damage site. So, pH2AX appears at damage sites in early stage, and is considered as a sensitive biomarker for DNA damage [37, 38]. We treated MEFs with 0.5mM MNU as reported [39], and detected pH2AX expression by immunofluorescence. Moreover, since comet assay is a rapid and sensitive method for detecting DNA damage in living cells [40, 41], we also analyzed DNA damage of MEFs cells by comet assay. The results showed delayed DNA repair in Fbxw7^+/−^ mice, suggesting that insufficiency of Fbxw7 disturbs the genomic stability and increases DNA damage. Generally, the damaged cells would result in apoptosis to maintain genomic stability. However, the cell apoptosis was decreased in Fbxw7^+/−^ mice, leading to the DNA damage accumulated (Figure 7). To our knowledge, our research firstly clarified the association of DNA damage with Fbxw7 haploinsufficiency based on MNU exposure. Haploinsufficiency of tumor suppressor, such as H2AX, Tip60 and BubR1, have also been reported to involve in genomic instability and accelerated tumorigenesis through direct or indirect participation in DNA damage repair [42–44]. Fbxw7 was reported to modulate genomic instability dependent on XRCC4 and cyclin E [12, 14]. It seems that Fbxw7 haploinsufficiency affects DNA damage repair through an indirect way. However, further studies are required to reveal the detailed mechanism behind this effect.

In summary, the present study demonstrates that Fbxw7 haploinsufficiency is associated with increased tumorigenesis in stomach, which may depend on dysregulated c-Myc degradation and DNA damage repair. The mouse model of Fbxw7 haploinsufficiency is a useful tool for gastric carcinogenesis study, especially for intestinal-type gastric cancer study.

## MATERIALS AND METHODS

### Mouse models

The ES cells harboring the mutant Fbxw7 allele were bought from European Conditional Mouse Mutagenesis Program (EUCOMM) (http://www.knockoutmouse.org/martsearch/project/27275) and transferred to the blastocoel cavities of 3.5 day blastocyst embryos. The embryos were transferred to surrogate mothers where gestation was completed. Then we crossed the chimeric founders with wild type C57BL/6 mice to generate heterozygous Fbxw7 knockout mice. Genomic DNA was extracted from mouse tail and assayed for the presence of Fbxw7-flox by polymerase chain reaction (PCR) with the following primers: loxP-F, 5′-GTTGAAATGCTTCGCTCGTTTGC-3′; loxP-R, 5′-CTGATGGCGAGCTCAGACCATAACT-3′; control-R, 5′-TGCCGTGTAACAGGCGTGCTAT-3′.

### Chemical-induced carcinogenesis by N-methyl-N-nitrosourea

The alkylating agent N-methyl-N-nitrosourea (MNU, Sigma, USA) was dissolved in distilled water at 240 mg/L, and freshly prepared 3 times per week for administration to the mice in drinking water in light-shielded bottles. 40 mice of 5 to 6 weeks old (20 Fbxw7^+/−^ mice and 20 Fbxw7^+/+^ mice) were used. These mice were exposed to MNU-contained drinking water every two weeks for a total of 5 weeks exposure. All mice were around 35 weeks of age at sacrifice.

### Protein expression detection of Fbxw7 and associated genes

The protein lysates were prepared from collected cells in lysis buffer containing 20 mM Hepes, 0.15 M NaCl and 1% Triton X-100 supplemented phosphatase inhibitor and protease inhibitor cocktail. Proteins were quantified by BCA, with bovine serum albumin as control. Proteins were separated by 10% sodium dodecyl sulfate polyacrylamide gel electrophoresis. PVDF membranes were incubated with the following antibodies: FBXW7-antibody (1:1000, Abcam, ab109617), c-Myc antibody (1:1000, Santa Cruz, 9e10), pH2AX antibody (1:1000, CST, #9718). GAPDH antibody was used for internal control.

### Detection of DNA damage on MEFs

MNU stock solution (50mM) was prepared freshly in dimethyl sulfoxide (DMSO) and diluted in PBS to a final DMSO concentration of 1%. Mouse embryo fibroblasts (MEFs) were extracted from E15.5d Fbxw7^+/−^ and Fbxw7^+/+^ mice and seeded in 6-well dishes (2 × 10^5^ cells/ml). After 24 h cultivation, the cells were exposed to 0.5 mM MNU in PBS for 30 min, and suspended in fresh growth medium. The comet assays were performed after 0 h, 2 h, 4 h, 8 h, 12 h, 18 h and 24 h of recovery at 37°C, with Trevigen Comet Assay^TM^ kit (Trevigen Inc., Gaithersburg, MD, USA). Cells were resuspended in ice cold PBS (Ca^2+^ and Mg^2+^ free) to a concentration of 1 × 10^5^ cells/ml. An aliquot of 50 μl of cells (1 × 10^5^ cells/ml) was added to 500 μl of molten LM agarose (1% low-melting agarose) kept at 42°C. Fifty microliters were immediately pipetted and evenly spread onto an area of the comet slides. The slides were incubated at 4°C in the dark for 10 min to accelerate gelling of the agarose disc and then transferred to prechilled lysis solution for 60 min at 4°C. A denaturation step was performed in 200 mM NaOH alkaline solution (1 mM EDTA, pH > 13) at room temperature for 20 min in the dark. The slides were subjected to electrophoresis at 1V/cm, 300 mA for 30 min in the dark at 4°C. At the end of the electrophoresis, the slides were washed with dH_2_O for 5 min, and immersed in70% ethanol for 5min and air dried. DNA was stained with 50 μl DAPI (1:1000 in Tris–EDTA buffer, pH 7.5) for 20 min in the refrigerator and immediately analyzed using fluorescence microscope. The tailDNA% and tail length were measured as DNA damage parameters. At least 50 cells were analyzed per sample.

### Immunohistochemistry and TUNEL

Mouse stomach was cut and unfolded along the greater curvature, and fixed in 4% formaldehyde solution. The 4 μm paraffin-embedded sections were deparaffinized and rehydrated for further immunohistochemical staining, with Ki67 antibody (1:100; Abcam) and Dako two-step detection Kit (Dako, CA, USA). The slides were counterstained with hematoxylin and viewed with light microscope. TUNEL assay was carried out according to the instructions (TUNEL Assay Kits for Apoptosis Detection, Roche). Five samples for each genotype and 10 fields per sample were observed

### Immunofluorescence

The MEFs cells (2 × 10^5^ cells/ml) from Fbxw7^+/−^ and Fbxw7^+/+^ mice were grown and treated in 24-well dishes. Following 24 h cultivation, the cells were exposed to 0.5 mM MNU in PBS for 30 min, and suspended in fresh growth medium. The cells were fixed in 4% paraformaldehyde and stained with pH2AX antibody (1:100, CST, #9718), and counterstained by DAPI. The slides were observed under the fluorescent microscope. At least 100 cells from each treatment condition were scored.

### Statistical analysis

The 2-tailed *X*^2^ test was used to determine the significance of the tumor incidence in each group. The non-parametric test was used to determine the significance of the difference between pathological scores of each group. The *X*^2^ or *T* test was used to determine the significance of pH2AX positive incidence and intensity. The *T* test was used to determine the significance of comet assay. In all tests, *P <* 0.05 was considered statistically significant. The SPSS software program (version 19.0; SPSS Inc., Chicago, IL, USA) was used for the statistical analysis.
